# Priorities for family physician and general practitioner recruitment and retention in Singapore: a PRIORITIZE study

**DOI:** 10.1186/s12875-021-01570-1

**Published:** 2021-11-16

**Authors:** Lorainne Tudor Car, Yee Sean Teng, Jin Wei How, Nadia Nasuha Binte Mohammad Nazri, Amy Li Xian Tan, Joanne Quah, Stephen Peckham, Helen Smith

**Affiliations:** 1 Lee Kong Chian School of Medicine, Nanyang Technological University, 11 Mandalay Road, Singapore, Singapore; 2 Department of Primary Care and Public Health, School of Public Health, Imperial College London, London, UK; 3 SingHealth Polyclinics, Singapore, Singapore; 4 Centre for Health Services Studies, University of Kent, Canterbury, UK

**Keywords:** Priority-setting, Primary care, Recruitment, Retention, Workforce, General practitioners, Family physicians

## Abstract

**Background:**

A shortage of primary care physicians has been reported in many countries. Primary care systems are diverse and the challenges leading to a decline in workforce are at times context-specific and require tailored solutions. Inviting frontline clinicians to share their insights can help identify optimal strategies for a particular setting. To determine priorities for family physicians’ and general practitioners’ recruitment and retention in Singapore, we invited primary care physicians to rank pertinent strategies using PRIORITIZE, a transparent, systematic priority-setting approach.

**Methods:**

The study advisory board, consisting of representatives of Singapore’s key primary care stakeholders, determined the criteria for prioritising of general practitioners (GPs) and family physicians (FPs) recruitment and retention strategies in Singapore. A comprehensive list of GPs and FPs recruitment and retention strategies was extracted from a recent systematic review of the relevant literature. A questionnaire listing the strategies and the scoring criteria was administered online to doctors practicing in public and private sector in Singapore. Respondents’ scores were combined to create a ranked list of locally most relevant strategies for improving GPs and FPs recruitment and retention.

**Results:**

We recruited a diverse sample of 50 GPs and FPs practicing in a variety of primary care settings, many with a range of additional professional responsibilities. Around 60 and 66% of respondents thought that there was a problem with recruitment and retention of GPs and FPs in Singapore, respectively. Strategies focusing on promoting primary care by emphasizing the advantages and enhancing the status of the profession as well as training-related strategies, such as sub-specialisation and high-quality rotations were considered priorities for improving recruitment. For retention of GPs and FPs, improving working conditions by increasing GPs’ and FPs’ salary and recognition, as well as varying or reducing time commitment, were seen as the most important strategies. The ranking between physicians working in public and private sector was mostly similar, with nine out of the top ten recruitment and retention strategies being the same.

**Conclusion:**

Primary care physicians’ ranking of recruitment and retention strategies for GPs and FPs in Singapore provide important insight into the challenges and the solutions as seen by the members of the profession themselves. This information can guide future policy and decision making in this area.

**Supplementary Information:**

The online version contains supplementary material available at 10.1186/s12875-021-01570-1.

## Background

Countries with person-centred and strong primary care have more efficient and equitable health care that leads to higher patient satisfaction and better outcomes [1]. Strong primary care can only be achieved with a competent primary care workforce. Yet many countries around the world report a shortage of primary care physicians, particularly in rural and underserved areas [2, 3]. This primary care workforce crisis can have a direct effect on the quality of provided care [4]. The reasons behind these shortages span issues relating to training, status, workload, additional responsibilities, working conditions and reductions in pay, but the importance of each often depends on the context [3].

Singapore, like many other developed countries worldwide, is faced with an ageing population and an increasing chronic disease burden [5]. Primary careis seen as crucial for accommodating these additional pressures to the healthcare system [6]. In Singapore, primary care is provided through government outpatient polyclinics and clinics run by private general practitioners (GPs). Polyclinics encompass several physicians and provide a comprehensive range of services for the family. Private GP clinics are solo practices that provide a more limited set of services. There are around 1700 private GP clinics and 20 polyclinics in Singapore. Private GPs clinics meet around 80% of primary care demand in Singapore, out of which only 20% is for chronic disease management [7] Singapore’s commitment to the strengthening of primary care workforce is reflected by the Health Care Manpower Plan 2020 which focuses on the improvement of family medicine clinics, community facilities and senior care centres. These changes are coupled with the introduction of Primary Care Networks, encouraging private clinics to work collaboratively and deliver more holistic care, as well as initiatives focused on primary care workforce development, e.g. postgraduate training opportunities [8–11].

Governments around the world have developed and implemented various policies aimed at addressing the problem of primary care physician recruitment and retention [4] . Primary care workforce challenges are often context-specific, and an understanding of the local setting is essential for the development of optimal strategies [12, 13]. As yet, little is known about the specific recruitment and retention interventions that could effectively strengthen the Singapore primary care workforce. Soliciting primary care clinicians’ views on optimal recruitment and retention strategies can assist in the development of effective policies given their unique insight into the local primary care training and practice. To this end, we invited general practitioners (GPs) and family medicine physicians (FPs) to prioritize recruitment and retention strategies for primary care physicians in Singapore using an established crowdsourcing, priority-setting approach.

## Methods

**Fig. 1:**
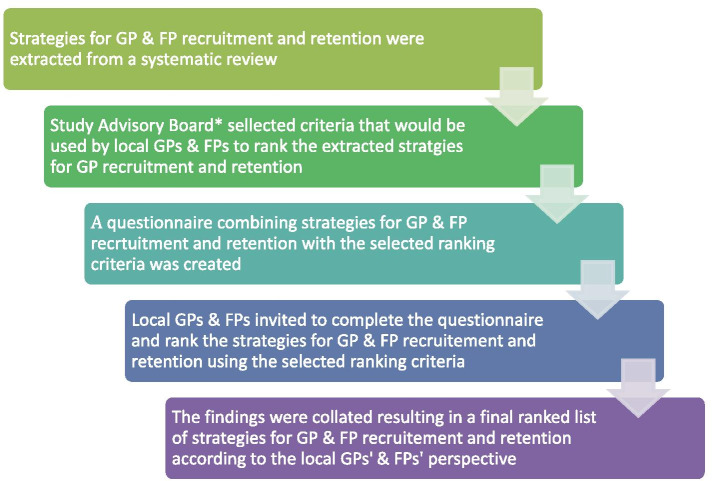
Modified PRIORITIZE methodology flow diagram. *Study advisory board consisted of Singapore Medical Association member, College of Family Physicians Singapore representative, Doctor trained but not spractising in Family Medicine, General Practitioner (Private solo), General Practitioner/Family Physician (Private group), Family Physician (Polyclinics), Medical Student, Program Director (Family Medicine Program for Residents) and a Ministry of Health representative

We implemented PRIORITIZE, an adaptation of the Child Health and Nutrition Research Initiative (CHNRI) methodology (Fig. 1) that has been used extensively to inform policymakers, funding bodies and international organizations about priorities for research [14, 15]. PRIORITIZE is a crowdsourcing, questionnaire-based priority-setting technique. When used to determine priorities in health care services delivery PRIORITIZE uses clinicians as experts. PRIORITIZE has been used to identify priorities for improvement of medication safety in primary care and care of people with cancer, prevention of delayed diagnosis in primary care as well as homecare safety of people with dementia [16–19]. We modified the PRIORITIZE methodology for this study (Fig. 1).

In previous PRIORITIZE exercises, we found that clinicians struggled to provide suggestions for priorities so instead, we extracted strategies for recruitment and retention of GPs and FPs from a pertinent and comprehensive systematic review of the literature by Marchand and Peckham [3]. Each strategy from the systemic review was studied in closer detail with reference to its primary literature and examined in terms of its rationale, implementation, and context. Strategies deemed irrelevant to Singapore’s healthcare context (e.g. those focusing on rural communities) were removedand discrepancies were resolved by discussion and consensus. Strategies that were considered relevant to the local context were further edited for clarity, as necessary. The final list of included 17 strategies focusing on recruitment and 16 on retention of GPs and FPs.

Strategies for improvement of GP and FP recruitment and retention were prioritised using relevant prioritization criteria. We compiled a list of different relevant criteria from previous PRIORITIZE and CHNRI studies and based on the discussions among the co-authors. We then invited the Study Advisory Board (SAB) to independently select up to five prioritization criteria in line with previous priority-setting studies. The SAB chose the following four prioritization criteria as the most relevant for evaluation of the strategies: Applicable locally: relevant to the local context

- Improves recruitment/retention: will improve recruitment/retention of GPs/FPs in Singapore
- Long term solution: can be a solution in the long term
- Doable: can be implemented in the local setting.

We then developed a questionnaire in English with a total of 33 suggestions for recruitment and retention, respectively and asked respondents to prioritize each one using the four criteria as in Box 1 (Additional file 1: Appendix 1). Data was collected on respondents’ demographics (gender, type of clinic, years in practice, overall hours of practice per week, other professional responsibilities). We also asked for respondents’ view on whether there is a problem with recruitment and retention of GPs and FPs in Singapore and quantified their perception of the severity of this problem on a scale of 0 to 10, where 0 represented no problem and 10 a major issue. The questionnaire was piloted on a sample of five primary care practitioners and was several strategies and prioritization criteria were amended in line with the received comments. For instance, the strategy ‘Increasing the length of time spent in general practice rotation in medical schools’ was presented together with the information on the duration of the family medicine rotations of each medical school in Singapore.

The final questionnaire was distributed on Qualtrics to primary care physicians working in Singapore via email lists and snowballing (i.e., participants were invited to forward the survey to their colleagues) [20]. Physicians working in both private and public primary care sector were eligible. The questionnaire was distributed to both general practitioners (i.e., physicians with a medical degree working in private primary care clinics) as well as family physicians (i.e., physicians who have undertaken postgraduate training in Family Medicine).

Respondents scored each recruitment and retention strategy according to the criteria using the following four options: ‘Yes - I agree’, ‘No - I do not agree’, ‘Unsure - I am unsure of whether or not I agree’, and ‘Unaware - I do not feel sufficiently familiar or confident to score this suggestion’. Each of the four options are awarded a score, ‘Yes’ - score 1, ‘No’ - score 0, ‘Unsure’ - score 0.5, and ‘Unaware’ - no score awarded. Enrolment was stopped once we had 50 complete responses as CHNRI simulations showing consistency of scores in groups of that size and bigger [21, 22].

We also categorised the strategies adapting the framework from the 10-point plan by the UK’s Department of Health and Social care, “Building the Workforce: the New Deal for General Practice” report which was also used in the Marchand and Peckham systematic review (Additional file 1: Appendix 2).

Intermediate scores i.e. scores for each criterion for every strategy were calculated by adding up the scores for all the responses (“1,” “0,” or “0.5”) and dividing the sum by the number of received answers. All intermediate scores were therefore assigned a value between 0 and 100. The overall priority score was then computed as the mean of the intermediate scores for each of the four criteria.. To determine the extent of agreement between the responses obtained we calculated the Average Expert Agreement (AEA) [14], that is the proportion of scorers who chose the mode (the most common score) for each research question using the following formula:

$$\mathrm{AEA}=\frac{1}{4}\sum \limits_{\mathrm{q}=1}^4\frac{\mathrm{N}\left(\mathrm{scorers}\ \mathrm{who}\ \mathrm{provided}\ \mathrm{the}\ \mathrm{most}\ \mathrm{frequent}\ \mathrm{response}\right)}{\mathrm{N}\left(\mathrm{scorers}\right)}$$

(where q is the strategy that clinicians are asked to evaluate to evaluate). We analysed the overall priority scores and AEA for the whole sample as well as separately for private and public physicians. We compared the ranking of strategies by private and public physicians descriptively.

This study was approved by the Nanyang Technological University Institutional Review Board (Reference Number: IRB-2018-12-020) and was conducted between Nov 2019 and Jun 2020 in Singapore. Informed consent was obtained from the participants in this study.

## Results

Of the 50 GP/FPs completing the questionnaire 30 were practicing in private clinics, 15 were from government polyclinics, three were practicing in community hospitals and two were locum physicians. 66% were male. Of 30 respondents from the private sector, 18 were from private group clinics and 12 were from private solo clinics. The median number of hours spent practicing (in any capacity) per week was 45, ranging from 12 to 100 h per week. Except for 11 respondents, all respondents had additional roles: 10 with teaching, administrative, and research roles, 6 both teaching and administrative, 14 administrative only, 8 teaching only and 1 research only. Other roles that were mentioned by respondents included business development, community and volunteer work and writing articles for a professional newsletter.

### Strategies for FPs and GPs recruitment

Most respondents (66%) thought that there was a problem with the recruitment of GPs and FPs in Singapore (Additional file 1: Appendix 3). Those responding “yes” were asked to quantify the severity of this problem on a scale 0 to 10. The median value of their responses was 7 (range 5–10).

The three highest ranked strategies for recruitment were related to GP lifestyle factors, enhanced status contribution of primary care practitioners and having sub-specialization in addition to their practice of family medicine (Table 1).

**Table 1:** The top ten strategies selected to improve recruitment of general practitioners and family physicians in Singapore

Rank	GPs and FPs recruitment strategy	Category	Priority score
1	Emphasizing GP/FPs’ lifestyle factors^a^	Promoting general practice	84.9
2	Enhancing status and contribution of primary care practitioners	Promoting general practice	84.7
3	Having sub-specialization and profiling of new skills	Improving breadth of training	82
4	Ensuring rotations are of high quality, with dedicated teaching faculty	Improving breadth of training	81.3
5	Emphasizing the holistic, community-oriented and patient-focused approach to family medicine	Promoting general practice	81.1
6	Having GP/FP role models	Promoting general practice	79.4
7	Modifying medical school curricula in primary care via exposure to varied patient settings	Improving breadth of training	78.8
8	Modifying medical school curricula in primary care via increased exposure to family medicine practice	Improving breadth of training	77.9
9	Enabling workplace experience and interaction with members of the profession	Improving breadth of training	77.2
10	Increasing and ensuring funding for fellowship training in primary care	Targeted financial support	75

^a^Emphasizing benefits of a GP/FP lifestyle such as flexibility, work-life balance or compatibility with family life

The two top ranked strategies, “Emphasizing GP/FPs lifestyle factors” and “Enhancing status contribution of primary care practitioners” were considered the most applicable to local context and most likely to improve recruitment. Strategies that were considered the easiest to implement in the local setting were “Emphasizing the holistic, community-oriented and patient-focused approach to family medicine” and “Enhancing status contribution of primary care practitioners”. Strategies that were most likely to be a long-term solution were “Enhancing status contribution of primary care practitioners” and “Having sub-specialization and profiling of new skills”.

Strategies that were considered the least important for GP recruitment were related to modification of undergraduate primary care education (Additional file 1: Appendix 4). These included increasing length of time spent in general practice rotation, establishing primary care honors and scholar tracks as well as developing fast-track programs.

### Strategies for FPs and GPs retention

Most respondents (60%) thought that there was a problem with the retention of GPs and FPs in Singapore (Additional file 1: Appendix 3). When asked to quantify the severity of this problem, the median value of their responses was 7 (range 4–10). The three top ranked strategies for retention of GPs and FPs were salary increase, increased recognition and varying time commitment across the working day and week.

In terms of the individual prioritization criteria, the two top strategies were also considered most applicable to Singapore, easiest to implement in the local setting, most likely to improve recruitment and most likely to be a long-term solution (Table 2). Strategies that were considered the least important for GP and FP retention in Singapore were “Establishing mentorship schemes” and “Reducing teaching responsibilities” (Additional file 1: Appendix 5).

**Table 2:** Top ten strategies to improve retention of general practitioners and family physicians in Singapore

Rank	GPs and FPs retention strategy	Category	Priority score
**1**	Increase GPs/FPs pay as an incentive to stay in the field	Improving working conditions	92
**2**	Increasing GPs/FPs recognition	Improving working conditions	90.5
**3**	Varying time commitment across the working day and week	New ways of working	87.4
**4**	Collaboration with colleagues from other specialties in managing complex patients	Collaboration	85.1
**5**	Establishing retainer schemes^a^ allowing for reduced working hours	Investment in retainer schemes	84.6
**6**	Reducing bureaucracy and practice administration work	Reducing other responsibilities	81.7
**7**	Enabling participation in part-time education posts or hospital attachment	New ways of working	81.1
**8**	Increasing job autonomy	New ways of working	80.1
**9**	Reducing management responsibilities	Reducing other responsibilities	78.2
**10**	Allowing for activities such as research and training in management skills	New ways of working	77.2

^a^ Retainer schemes were developed by the UK Department of Health to enable doctors who can only take on a limited amount of clinical work to stay in practice, retain their skills and progress their careers with a view to returning to NHS general practice

### Perspectives from private and public doctors

When comparing ranking of strategies between physicians working in private and public sector (Additional file 1: Appendix 6 and 7), nine out of the top 10 recruitment and retention strategies were the same between these two groups of respondents. The ranking of the strategies overall for both recruitment and retention was largely similar except for six strategies for recruitment and five strategies for retention which were ranked differently by each group (Additional file 1: Appendix 6 and 7). For recruitment, respondents from the public sector felt that “Emphasizing GP/FPs’ lifestyle factors” and “Ensuring rotations are of high quality, with dedicated teaching faculty” (ranked 1st and 2nd^,^ respectively) were more important than their colleagues from private sector (ranked 6th and 8th respectively). Conversely, “Modifying medical school curricula in primary care via increased exposure to family medicine practice” was seen more important for recruitment by the private (ranked 2nd) than the public sector physicians (ranked 10th).

For retention, “Reducing bureaucracy and practice administration” and “Establishing social support initiatives to enhance relationships and collaboration with colleagues” were considered more important among the private (ranked 5th and 8th, respectively) then the public sector physicians (ranked 9th and 13th, respectively). Conversely, “Reducing management responsibilities” and “Allowing for activities such as research and training in management skills” were considered more important by the public (ranked 4th and 7th, respectively) than the private sector physicians (ranked 10th and 15th, respectively).

The highest ranked strategies had the highest AEA, which shows that there was a stronger consensus among GP/FPs for the top ranked strategies compared to those ranked lower (Additional file 1: Appendix 3 and 4). This was observed for both recruitment and retention strategies. Those ranked lower which had a significant number of “Unsure” and “Unaware” answers to scoring.

## Discussion

### Summary

Most primary care physicians thought that there was a problem with recruitment and retention of GPs and FPs in Singapore. Strategies focusing on promoting general practice by emphasizing its advantages and enhancing its status, as well as by improving training through sub-specialisation and high-quality rotations were considered priorities for improving recruitment of GPs and FPs. For retention of GPs and FPs, improving working conditions such as an increase in salary, public recognition and varying or reducing time commitment were considered the most important strategies.

### Comparison with the existing literature

Prioritization in primary care involving primary care physicians mostly focuses on resource allocation [23, 24]. Some studies involve diverse stakeholders including primary care physicians in priority setting in primary care research [25]. In addition to these important objectives, Decision-makers at the institutional, regional, and national level should harness front-line clinicians’ insight to improve the quality, safety, and efficiency of healthcare delivery. There are examples of similar initiatives through focus group discussions or surveys [26, 27]. To our knowledge, PRIORITIZE is the only approach to date that uses a systematic, priority setting approach to this end [17, 18]. In this study, we used a modified PRIORITIZE approach to identify local frontline clinicians’ views on recruitment and retention. We were unable to find a similar study prioritizing GP recruitment and retention strategy in other settings.

The literature on recruitment and retention of GPs, mostly originates from high-income countries, such as UK or the US, and is largely in form of qualitative research or surveys without prioritization [3, 28–30]. Marchand’s and Peckham’s systematic review analysed findings from 36 studies on recruitment and retention from diverse high-income, mostly English-speaking countries. In terms of recruitment, this systematic review extracted diverse strategies from the original studies but concluded that strategies addressing intrinsic recruitment factors such as receiving recognition and providing varied and continuous provision of patient care were more important than extrinsic factor such as loan forgiveness [3]. This is similar to our findings showing that local GPs and FPs considered recruitment strategies focused on enhancing status and improving the breadth of training rather than targeted financial support as more important. In terms of other local evidence, a recent qualitative study exploring medical students’ attitudes towards careers in primary care in Singapore identified several important and potentially detrimental factors [31]. These included limited professional opportunities, emphasis on lifestyle benefits rather than professional characteristics, need for business acumen, conflicts created by business in clinical care, mundane case mix, lack of continuity of care, limited consultation time, and specialists’ negative attitudes towards family doctors. Recruitment strategies such as “Emphasizing the holistic, community-oriented and patient-focused approach to family medicine” and “Enhancing status contribution of primary care practitioners”, which were prioritised by the respondents in our study, could help to address some of these concerns. Interestingly, Singaporean medical students felt that putting an emphasis on the life-style balance, which was the top recruitment strategy as seen by the local GPs and FPs, without focusing on the professional aspect or impact on care may deter some student from choosing a career in family medicine.

Retention strategies prioritised in this study are comparable with the other research in this area which mostly originate from high-income, western countries. A cross sectional study exploring motivation for career choice and job satisfaction among GP trainees and newly qualified GPs in seven European countries identified compatibility with family life and general practice being a challenging medically broad discipline as the main motivators for choosing a career in general practice [28]. It also showed a significant correlation between workload and mean income and the level of satisfaction. Correspondingly, our respondents felt that an emphasis on life-work balance and holistic, community-oriented, and patient-focused approach were top strategies to improve recruitment while increasing pay and varying time commitment were important for retention. Furthermore, a systematic review exploring factors determining GP satisfaction in clinical practice showed that flexible workload and receiving recognition, collaboration with colleagues from other specialities and engaging in other areas such as research were important [32]. Similarly, in our study strategies relating to recognition, flexible workload, and collaboration with colleagues from other specialities were ranked second, third and fourth, respectively. Furthermore, while emphasizing benefits of a GP lifestyle was seen as important for recruitment, for retention having more flexible working hours was seen as a top priority. This apparent duality may mean that the current working arrangement of GPs, although enabling greater work-life balance compared to other specialities, could be made even more flexible to improve retention.

### Implication for future practice and research

Ranking of strategies for recruitment and retention of GPs and FPs as seen by practicing primary care clinicians provides important insights that can inform future policy making. Some of the top ranked strategies may be easily implementable and could be taken into consideration by educators and decision-makers in Singapore. A number of top strategies for recruitment focused on the promotion of a career in general practice. This can be achieved through initiatives that generate respect amongst doctors as well as society at large. Recruitment strategies that were ranked high and may be achievable in the local setting are those focusing on education at both undergraduate (e.g., ensuring high quality rotation and increased exposure to family medicine) and postgraduate level (e.g., a possibility of sub-specialisation). Notably, educational strategies which may be more difficult to introduce such as increasing length of general practice rotations or introducing educational tracks and programs focusing exclusively on general practice were not considered a priority. Retention-related strategies that were seen as important and could be considered for implementation are those enabling new ways of working through varying time commitment, enabling part-time hospital attachments and allowing for engagement in research or management, particularly by physicians working in public sector. In addition, we note some important differences between the private and public sector physicians in terms of priorities for recruitment and retention. For recruitment, private GPs felt found increase exposure to family medicine in medical school much more important than public sector physicians. For retention, both groups called for reduction of non-clinical responsibilities; administration-related among private GPs and management-related ones among public GPs. This seems to indicate a need for additional, specific non-clinical support in both sectors. Furthermore, social support from colleagues was seen as more important by private GPs, who mostly work in solo practices. Conversely, public sector physicians found involvement in research and additional training important for retention unlike private GPs who may have easier access to such opportunities.

Existing evidence on effectiveness of strategies for recruitment and retention is limited. To address this gap, it is important that the future implementation of such strategies is accompanied by robust evaluation. In addition, future research should explore additional strategies specific to Singapore which may have not been covered in the existing literature. It should also aim to investigate in more depth different priorities for private and public sector. Finally, to inform national strategy for requitement and retention of GPs in Singapore, there is a need for studies that would explore views form other stakeholders such as medical educators and policy makers.

### Limitations & strengths

Our study included a diverse group of primary care physicians working in the private and public sector in Singapore. Our respondents were often involved in a number of other professional responsibilities and worked in different primary care settings. We used an established priority-setting approach which was previously used in other settings and disciplines. However, we modified it to minimize the burden on the clinicians an boost the rate of recruitment. This was done by extracting strategies for prioritization from a comprehensive systematic review instead of inviting physicians to volunteer strategies themselves. We aimed to avoid low response rates and an extension to the project duration observed in previous PRIORITIZE exercises. There are also some limitations to our study. While we extracted strategies from a comprehensive systematic review that collated evidence from diverse settings, there may be some additional strategies specific to Singapore context which were not mentioned. Our questionnaire did not include open-ended questions inviting additional suggestions for prioritization strategies from the clinicians. In addition, CHNRI simulation studies show that the ranking scores in this priority-setting approach remain similar upwards of 50 respondents. However, primary care physician workforce in Singapore is much larger and diverse. Future studies with a larger sample of primary care physicians would allow for more comparative analyses between the private and public sectors.

## Conclusion

In our study, Singaporean primary care physicians ranked GPs and FPs recruitment and retention strategies in Singapore. Top strategies for recruitment focused on promoting general practice as a career and improving the breadth of training. Top strategies for retention focused on improving working conditions, such as pay and status, as well as offering new, more flexible ways of working. This ranking of recruitment and retention strategies by the local frontline clinicians provides important insight into the challenges and the solutions as seen by the members of the professions themselves. Our findings can help inform future policy and decision making in this area.

## Supplementary Information

**Additional file 1: Appendix 1.** Excerpt from the Qualtrics questionnaire showing strategies and ranking criteria. **Appendix 2.** Framework for classification of recruitment and retention strategies for general practitioners and family physicians in Singapore adapted from "Building the Workforce — the New Deal for General Practice” by the UK Department of Health and “Addressing the crisis of GP recruitment and retention: a systematic review” by Marchand and Peckham. **Appendix 3.** Respondents’ views on GPs and FPs recruitment and retention problems in Singapore. **Appendix 4.** Ranking of strategies to boost GPs and FPs recruitment. **Appendix 5.** Ranking of strategies for GPs and FPs retention. **Appendix 6.** Comparison of rankings by private and public sector physicians relating to the strategies to boost recruitment of GPs and FPs. **Appendix 7.** Comparison of ranking Comparison of rankings by private and public sector physicians relating to the strategies to boost retention GPs and FPs.

## Data Availability

The datasets used and/or analysed during the current study are available from the corresponding author on reasonable request.
