# Supplementary material for: Priorities for family physician and general practitioner recruitment and retention in Singapore: a PRIORITIZE study
Source: BMC Fam Pract. 2021 Nov 16;22:229. doi: 10.1186/s12875-021-01570-1 (PMC8596925; doi:10.1186/s12875-021-01570-1)
Supplement: Supplementary file 1 — Additional file 1: Appendix 1. Excerpt from the Qualtrics questionnaire showing strategies and ranking criteria. Appendix 2. Framework for classification of recruitment and retention strategies for general practitioners and family physicians in Singapore adapted from "Building the Workforce — the New Deal for General Practice” by the UK Department of Health and “Addressing the crisis of GP recruitment and retention: a systematic review” by Marchand and Peckham. Appendix 3. Respondents’ views on GPs and FPs recruitment and retention problems in Singapore. Appendix 4. Ranking of strategies to boost GPs and FPs recruitment. Appendix 5. Ranking of strategies for GPs and FPs retention. Appendix 6. Comparison of rankings by private and public sector physicians relating to the strategies to boost recruitment of GPs and FPs. Appendix 7. Comparison of ranking Comparison of rankings by private and public sector physicians relating to the strategies to boost retention GPs and FPs. [file 12875_2021_1570_MOESM1_ESM.docx]

# **Priorities for family physician and general practitioner recruitment and retention in Singapore: a PRIORITIZE study**

# Appendix

**Appendix 1.** Excerpt from the Qualtrics questionnaire showing strategies and ranking criteria


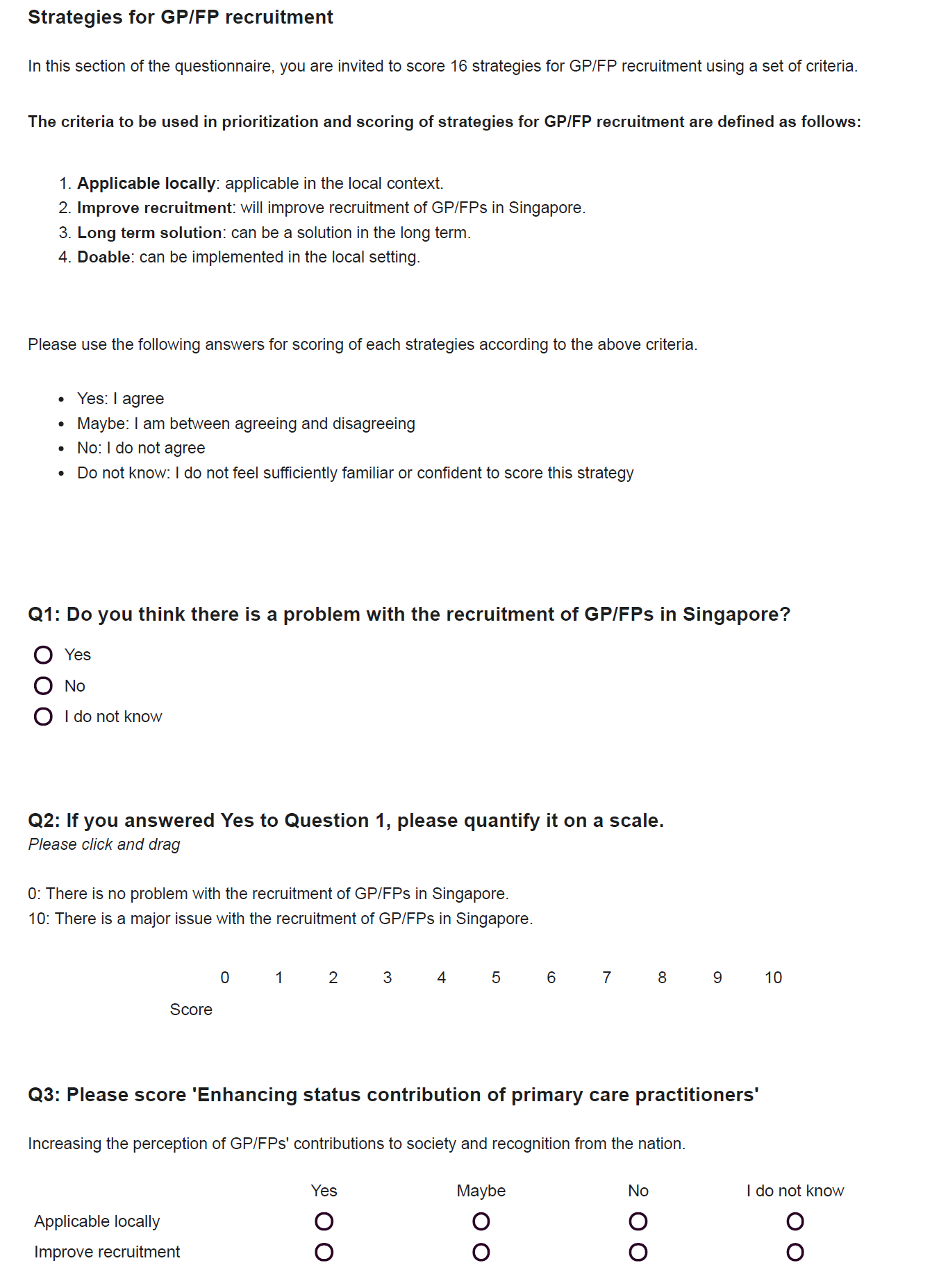


**
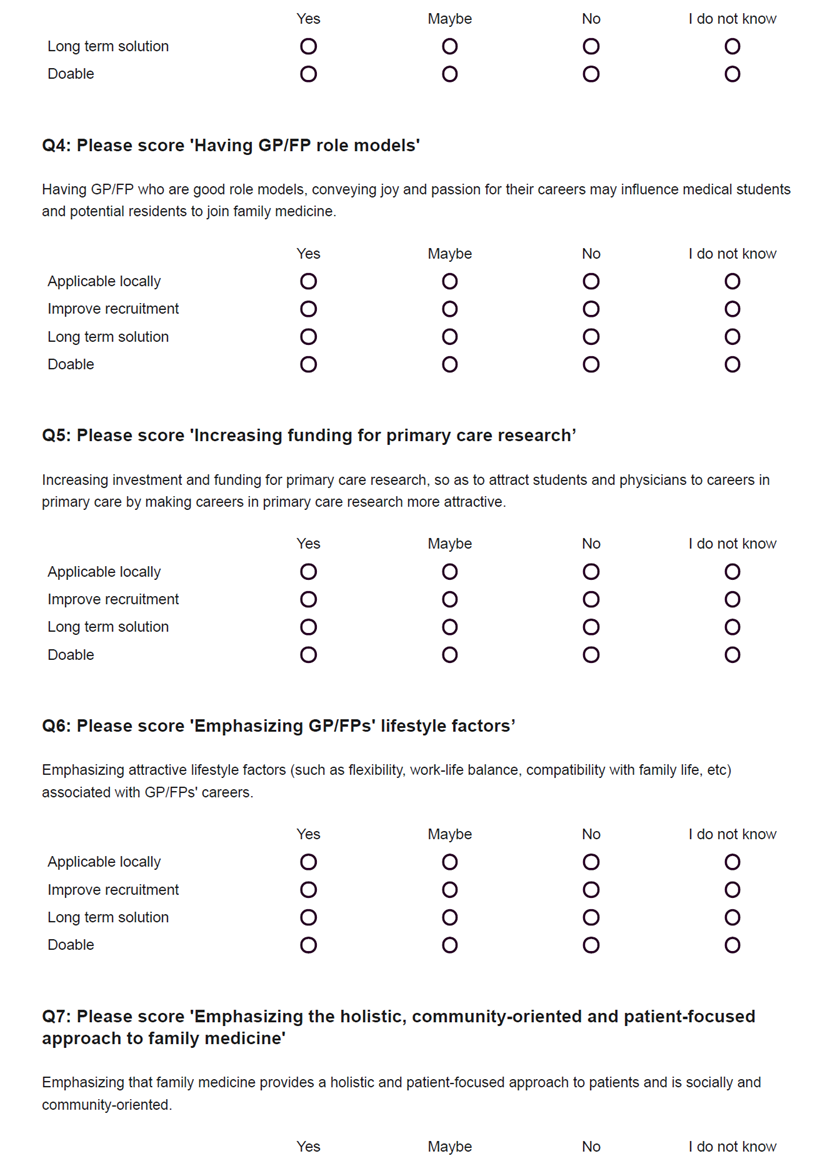
**

**Appendix 2.** Framework for classification of recruitment and retention strategies for general practitioners (GPs) and family physicians (FPs) in Singapore adapted from 10-point plan from Building the Workforce — the New Deal for General Practice” developed by the UK Department of Health and systematic review and “Addressing the crisis of GP recruitment and retention: a systematic review” by Marchand and Peckham (reference)

| **Recruitment** | 1. Promoting general practice | - Enhancing the status, contribution, career advancement, and rewards of primary care practitioners - Role models - Medical environment important |
| --- | --- | --- |
|  | 2. Improving the breadth of training | *Exposure to general practice:*   - Early exposure/pre-registration house officer scheme - Workplace experience and interaction with members of the profession - Length of time spent in general practice rotation - Ensuring that the rotations are of high quality, with dedicated generalists’ faculty   *Curricula modifications:*   - Effective medical school curricula in primary care - Establish primary care honours or scholars’ tracks - Develop or expand primary care fast-track programmes - Subspecialisation, portfolio careers, and profile of new skills   *Recruitment/admission:*   - Modification of selection criteria |
|  | 3. Targeted financial support | - Link choice of career in primary care to loan forgiveness - Funding in primary care research - Increase and assure funding for fellowship training in primary care - Direct training funds to schools with track records of producing graduates in primary care |
| **Retention** | 4. Investment in retainer schemes* | *Widening the scope of remuneration and contract conditions:*  · Reduce the income differential between general practice and hospital work   - Remove the disincentives for less than full-time employment, widening of the employment mechanisms open to GPs, such as authority-organised salaried schemes |
|  | 5. Improving the training capacity | Sub-specialization and portfolio careers where doctors might gain skills in a range of specialties and practices, some or all of them at any one time |
|  | 6. Improving working conditions | · Increase in salary  · Increase in recognition |
|  | 7. New ways of working | *Varying time commitment across the working day and week:*   - Part time, job share, temporary, and short time available, GP’s employment status and career stage   *Offering a wider choice of long-term career paths:*   - Locum and associate positions equal to full-time principal posts - Activities such as research and training in management skills - A part-time educational post or hospital attachment - Job mobility as a way to progress (a more positive vision of mobility) - Increased job autonomy |
|  | 8. Reducing other responsibilities | Reducing administrative, teaching, managerial etc. workload |
|  | 9. Collaboration with colleagues | Social support, relationship, and collaboration with colleagues/patients |

**Appendix 3.** Respondents’ views on GPs and FPs recruitment and retention problems in Singapore

**Appendix 4.** Ranking of strategies to boost GPs and FPs recruitment

| **Rank** | **GPs and FPs recruitment strategy** | **Recruitment strategy category** | **Priority score** | **Average Expert Agreement (AEA)*** |
| --- | --- | --- | --- | --- |
| **1** | Emphasizing GP/FPs' lifestyle factors | Promoting general practice | 84.9 | 0.74 |
| **2** | Enhancing status contribution of primary care practitioners | Promoting general practice | 84.7 | 0.71 |
| **3** | Having sub-specialization and profiling of new skills | Improving breadth of training | 82 | 0.69 |
| **4** | Ensuring rotations are of high quality, with dedicated teaching faculty | Improving breadth of training | 81.3 | 0.64 |
| **5** | Emphasizing the holistic, community-oriented and patient-focused approach to family medicine | Promoting general practice | 81.1 | 0.66 |
| **6** | Having GP/FP role models | Promoting general practice | 79.4 | 0.65 |
| **7** | Modifying medical school curricula in primary care via exposure to varied patient settings | Improving breadth of training | 78.8 | 0.63 |
| **8** | Modifying medical school curricula in primary care via increased exposure to family medicine practice | Improving breadth of training | 77.9 | 0.63 |
| **9** | Enabling workplace experience and interaction with members of the profession | Improving breadth of training | 77.2 | 0.58 |
| **10** | Increasing and ensuring funding for fellowship training in primary care | Targeted financial support | 75 | 0.57 |
| **11** | Linking choice of career in primary care to loan forgiveness | Targeted financial support | 72.8 | 0.58 |
| **12** | Establishing early exposure via Student Assistantship or Internship Programmes | Improving breadth of training | 70.9 | 0.50 |
| **13** | Increasing funding for primary care research | Targeted financial support | 69.9 | 0.52 |
| **14** | Directing training funds to schools with track records of producing graduates in primary care | Targeted financial support | 67.9 | 0.48 |
| **15** | Increasing the length of time spent in general practice rotation in medical schools | Improving breadth of training | 67.3 | 0.49 |
| **16** | Establishing primary care honors or scholar tracks | Improving breadth of training | 65.4 | 0.48 |
| **17** | Developing or expanding primary care fast-track programs | Improving breadth of training | 58.8 | 0.42 |

*Average expert agreement (AEA) values range from 0.00 to 1.00

**Appendix 5.** Ranking of strategies for GPs and FPs retention

| **Rank** | **GPs and FPs retention strategy** | **Retention strategy category** | **Priority score** | | **Average Expert Agreement (AEA)*** |
| --- | --- | --- | --- | --- | --- |
| **1** | Increase GP/FPs pay as as incentive to stay in the field | Incentives to remain in practice | | 92 | 0.84 |
| **2** | Increasing GP/FP recognition | Promoting general practice | | 90.5 | 0.81 |
| **3** | Varying time commitment across the working day and week | New ways of working | | 87.4 | 0.78 |
| **4** | Collaboration with colleagues from other specialties in managing complex patients | Collaboration with colleagues | | 85.1 | 0.72 |
| **5** | Establishing retainer schemes allowing for reduced working hours | Investment in retainer schemes | | 84.6 | 0.71 |
| **6** | Reducing bureaucracy and practice administration work | Reducing other responsibilities | | 81.7 | 0.65 |
| **7** | Enabling participation in part-time education posts or hospital attachment | New ways of working | | 81.1 | 0.71 |
| **8** | Increasing job autonomy | New ways of working | | 80.1 | 0.61 |
| **9** | Reducing management responsibilities | Reducing other responsibilities | | 78.2 | 0.61 |
| **10** | Allowing for activities such as research and training in management skills | New ways of working | | 77.2 | 0.62 |
| **11** | Sub-specialization where doctors might gain skills in a range of specialties and practices, some or all of them at any one time | Improving the training capacity | | 76.9 | 0.65 |
| **12** | Offering a wider choice of long-term career paths by considering locum positions equal to full-time principal posts | New ways of working | | 76.7 | 0.61 |
| **13** | Establishing social support initiatives to enhance relationships and collaboration with colleagues | Collaboration with colleagues | | 76.2 | 0.61 |
| **14** | Academic hospital and centers teaching medical students and advanced students | New ways of working | | 72.5 | 0.50 |
| **15** | Establishing mentorship schemes | Improving the training capacity | | 72.1 | 0.55 |
| **16** | Reducing teaching responsibilities | Reducing other responsibilities | | 52.3 | 0.43 |

. *Average expert agreement (AEA) values range from 0 to 1

**Appendix 6.** Comparison of rankings by private and public sector physicians relating to the strategies to boost recruitment of GPs and FPs*

| Private | Public |
| --- | --- |
| 1. Enhancing status contribution of primary care practitioners | 1. Emphasizing GP/FPs' lifestyle factors |
| 1. Modifying medical school curricula in primary care via increased exposure to family medicine practice* | 1. Ensuring rotations are of high quality, with dedicated teaching faculty |
| 1. Emphasizing the holistic, community-oriented and patient-focused approach to family medicine | 1. Having sub-specialization and profiling of new skills |
| 1. Having sub-specialization and profiling of new skills | 1. Enhancing status contribution of primary care practitioners |
| 1. Having GP/FP role models | 1. Having GP/FP role models |
| 1. Emphasizing GP/FPs' lifestyle factors | 1. Emphasizing the holistic, community-oriented and patient-focused approach to family medicine |
| 1. Modifying medical school curricula in primary care via exposure to varied patient settings | 1. Modifying medical school curricula in primary care via exposure to varied patient settings |
| 1. Ensuring rotations are of high quality, with dedicated teaching faculty | 1. Enabling workplace experience and interaction with members of the profession |
| 1. Enabling workplace experience and interaction with members of the profession | 1. Increasing and ensuring funding for fellowship training in primary care |
| 1. Linking choice of career in primary care to loan forgiveness | 1. Modifying medical school curricula in primary care via increased exposure to family medicine practice |
| 1. Increasing funding for primary care research | 1. Linking choice of career in primary care to loan forgiveness |
| 1. Increasing and ensuring funding for fellowship training in primary care | 1. Establishing early exposure via Student Assistantship or Internship Programmes |
| 1. Directing training funds to schools with track records of producing graduates in primary care | 1. Establishing primary care honour or scholar tracks |
| 1. Establishing early exposure via Student Assistantship or Internship Programmes | 1. Increasing the length of time spent in general practice rotation in medical schools |
| 1. Increasing the length of time spent in general practice rotation in medical schools | 1. Increasing funding for primary care research |
| 1. Establishing primary care honour or scholar tracks | 1. Directing training funds to schools with track records of producing graduates in primary care |
| 1. Developing or expanding primary care fast-track programs | 1. Developing or expanding primary care fast-track programs |

*Strategies highlighted in yellow had distinctly different ranking (defined as being more than four places apparat) between private and public sector physicians

**Appendix 7.** Comparison of ranking Comparison of rankings by private and public sector physicians relating to the strategies to boost retention GPs and FPs*

| Private | Public |
| --- | --- |
| 1. Increase GP/FPs pay as as incentive to stay in the field | 1. Varying time commitment across the working day and week |
| 1. Increasing GP/FP recognition | 1. Increase GP/FPs pay as as incentive to stay in the field |
| 1. Collaboration with colleagues from other specialties in managing complex patients | 1. Establishing retainer schemes allowing for reduced working hours |
| 1. Varying time commitment across the working day and week | 1. Reducing management responsibilities |
| 1. Reducing bureaucracy and practice administration work* | 1. Collaboration with colleagues from other specialties in managing complex patients |
| 1. Establishing retainer schemes allowing for reduced working hours | 1. Increasing GP/FP recognition |
| 1. Increasing job autonomy | 1. Allowing for activities such as research and training in management skills |
| 1. Establishing social support initiatives to enhance relationships and collaboration with colleagues | 1. Enabling participation in part-time education posts or hospital attachment |
| 1. Enabling participation in part-time education posts or hospital attachment | 1. Reducing bureaucracy and practice administration work |
| 1. Reducing management responsibilities | 1. Increasing job autonomy |
| 1. Academic hospital and centers teaching medical students and advanced students | 1. Sub-specialization where doctors might gain skills in a range of specialties and practices, some or all of them at any one time |
| 1. Sub-specialization where doctors might gain skills in a range of specialties and practices, some or all of them at any one time | 1. Offering a wider choice of long-term career paths by considering locum positions equal to full-time principal posts |
| 1. Offering a wider choice of long-term career paths by considering locum positions equal to full-time principal posts | 1. Establishing social support initiatives to enhance relationships and collaboration with colleagues |
| 1. Establishing mentorship schemes | 1. Establishing mentorship schemes |
| 1. Allowing for activities such as research and training in management skills | 1. Academic hospital and centers teaching medical students and advanced students |
| 1. Reducing teaching responsibilities | 1. Reducing teaching responsibilities |

*Strategies highlighted in yellow had markedly different ranking defined as being more than four places apparat) between private and public sector physicians
